# Morphology of en face Haller vessel and macular neovascularization at baseline and 3 months as predictive factors in age-related macular degeneration

**DOI:** 10.1038/s41598-022-15139-0

**Published:** 2022-06-25

**Authors:** Hyungwoo Lee, SoHyeon Kim, Myung Ae Kim, Hyewon Chung, Hyung Chan Kim

**Affiliations:** Department of Ophthalmology, Konkuk University Medical Center, Konkuk University School of Medicine, 120-1 Neungdong-ro, Gwangjin-gu, Seoul, 05030 Republic of Korea

**Keywords:** Retinal diseases, Eye diseases

## Abstract

The clinical implication of en face imaging of Haller vessels and macular neovascularization (MNV) in neovascular age-related macular degeneration (nAMD) is not well established. The purpose of this study is to investigate whether the early-phase morphology of en face Haller vessel and MNV is related to the injection frequency and visual outcome in treatment-naïve nAMD. En face images of Haller vessel and MNV were acquired from 52 eyes at baseline, after three loading doses and at 12 months later using optical coherence tomography (OCT) and OCT angiography. Vessel area, diameter, length, intersection number, fractal dimension, and lacunarity were calculated. Patients were classified according to the injection frequency (< 5 as the infrequent group) and visual gain (≥ 0.3 logMAR) over 12 months. The infrequent group was associated with a longer Haller vessel length after loading doses (OR 3.05, P = 0.01), while visual gain was associated with a smaller maximal MNV diameter after loading doses (OR 0.22, P = 0.03). A predictive model for frequent injection based on the Haller vessel length demonstrated an AUC of 0.71. In conclusion, the en face Haller vessel and MNV morphology after loading doses can be used as biomarkers for the injection frequency and visual gain during the first year in treatment-naïve nAMD patients.

## Introduction

Neovascular age-related macular degeneration (nAMD) is a leading cause of blindness in developed countries, and anti-vascular endothelial growth factor (VEGF) is the mainstay treatment^1^. The introduction of optical coherence tomography (OCT) and OCT angiography (OCTA) has enabled the detailed analysis of macular neovascularization (MNV), the key pathological event leading to nAMD^2^. MNV morphology on OCTA images has been shown to reflect disease activity^3–6^. In particular, the quantitation of parameters, such as area, density, complexity, number of intersections, and vessel diameter, can determine the extent of morphologic changes, which is an advantage over qualitative analyses^3,4,6^. Most studies to date have investigated cross-sectional data of treated patients, whereas the prognostic values of early morphologic MNV changes (both at baseline and after loading doses) in treatment-naïve patients with nAMD have not been well studied.

Haller vessel morphology and clinical implications thereof have recently been investigated. As MNV is a choroidal event, examining the choroid itself together with MNV might be important to understand the pathology of nAMD. Pachyvessels—dilated Haller vessels—are believed to be closely associated with choriocapillaris atrophy and the development of focal disruptions in the retinal pigment epithelium and Bruch’s membrane, leading to MNV development^7^. Studies using enhanced depth imaging OCT have shown that pachyvessels are observed in typical AMD and polypoidal choroidal vasculopathy (PCV)^8,9^. Compared to enhanced depth imaging OCT, which shows the vertical cross-section of the choroid, en face OCT imaging has the advantage of exposing the detailed two-dimensional morphology of the Haller vessel in a single image, enabling the quantification of the distribution vessel diameter, area and length, and an analysis of vessel complexity^10,11^. These vascular characteristics might influence the localized flow and mural pressure of Haller vessels, the overlying choriocapillaris and MNV. En face Haller imaging also has the advantage of easy acquisition because it is performed in conjunction with OCTA imaging. However, the early prognostic value of en face Haller vessel morphology in patients with nAMD is not well understood. Furthermore, integrative analyses considering both MNV and en face Haller vessel morphologies have not yet been well studied.

Predicting the optimal treatment interval and vision-related prognosis is important to avoid undertreatment, and identifying the optimal treatment during the first 12 months is crucial for determining long-term prognosis^12,13^. Therefore, in this study, we aimed to quantify the early morphologies of both the Haller vessel and MNV using en face OCT and OCTA, respectively, in treatment-naïve patients with nAMD at the early loading phase and to determine their association with the injection frequency and visual outcomes after 12 months. Comprehensive quantification of Haller vessel and MNV morphologies was performed to obtain vessel diameter, area and length and analyze network structures, including the number of intersections, fractal dimension (FD) and lacunarity. We then investigated whether the quantified factors were associated with any prognostic significance in terms of visual acuity changes and the number of treatments during the first 12 months.

## Results

### Baseline characteristics

A total of 52 eyes from 51 consecutive patients with a mean age of 70.7 ± 8.4 years (range, 51–86 years) were included in this study. Twenty-nine of the 81 eyes that were initially screened were excluded because of combined epiretinal membrane (N = 4), poor image quality due to progressed cataract (N = 7), and poor image quality on the OCTA and/or structural en face OCT images (N = 18). The numbers of eyes with typical nAMD and PCV were 33 and 19, respectively. The mean subfoveal choroidal thickness (SFCT) in typical nAMD and PCV was 242.9 ± 116.4 μm and 300.2 ± 75.7 μm, respectively. Aflibercept and ranibizumab were administered to 42 and 10 eyes, respectively. The mean number of intravitreal injections (IVIs) during the first 12 months was 5.0 ± 1.6. The mean logMAR visual acuity was 0.56 ± 0.57 at baseline and 0.38 ± 0.36 after 12 months.

The baseline demographic and clinical characteristics of the ‘infrequent’ and “frequent’ subgroups and those of the ‘visual gain’ and ‘no visual gain’ subgroups are summarized in Supplementary Table 1. Between subgroups divided by the injection frequencies, the ‘infrequent’ injection group had higher ratio of female and better visual acuity after 12 months. Between subgroups divided by the visual gain, the ‘visual gain’ group had poorer visual acuity at baseline and after loading doses. The ratio of PCV to typical nAMD and SFCT were not different between subgroups classified by injection frequencies. Subgroups classified by visual gain also did not show any significant differences in regard to the ratio of PCV to typical nAMD and SFCT.

### Mean differences in Haller vessel and MNV parameters according to the injection number and visual gain

The eyes in the infrequent group had a longer total Haller vessel length after three loading doses, a higher number of Haller vessel intersections at 12 months, an increased Haller vessel FD at 12 months, and a shorter MNV branch length (mean and SD) at baseline than those of the frequent group (Supplementary Table 2). There was no significant difference in either Haller or MNV parameters between the visual gain and no visual gain groups (Supplementary Table 3).

### Haller vessel and MNV parameters associated with injection number

**Figure 1:**
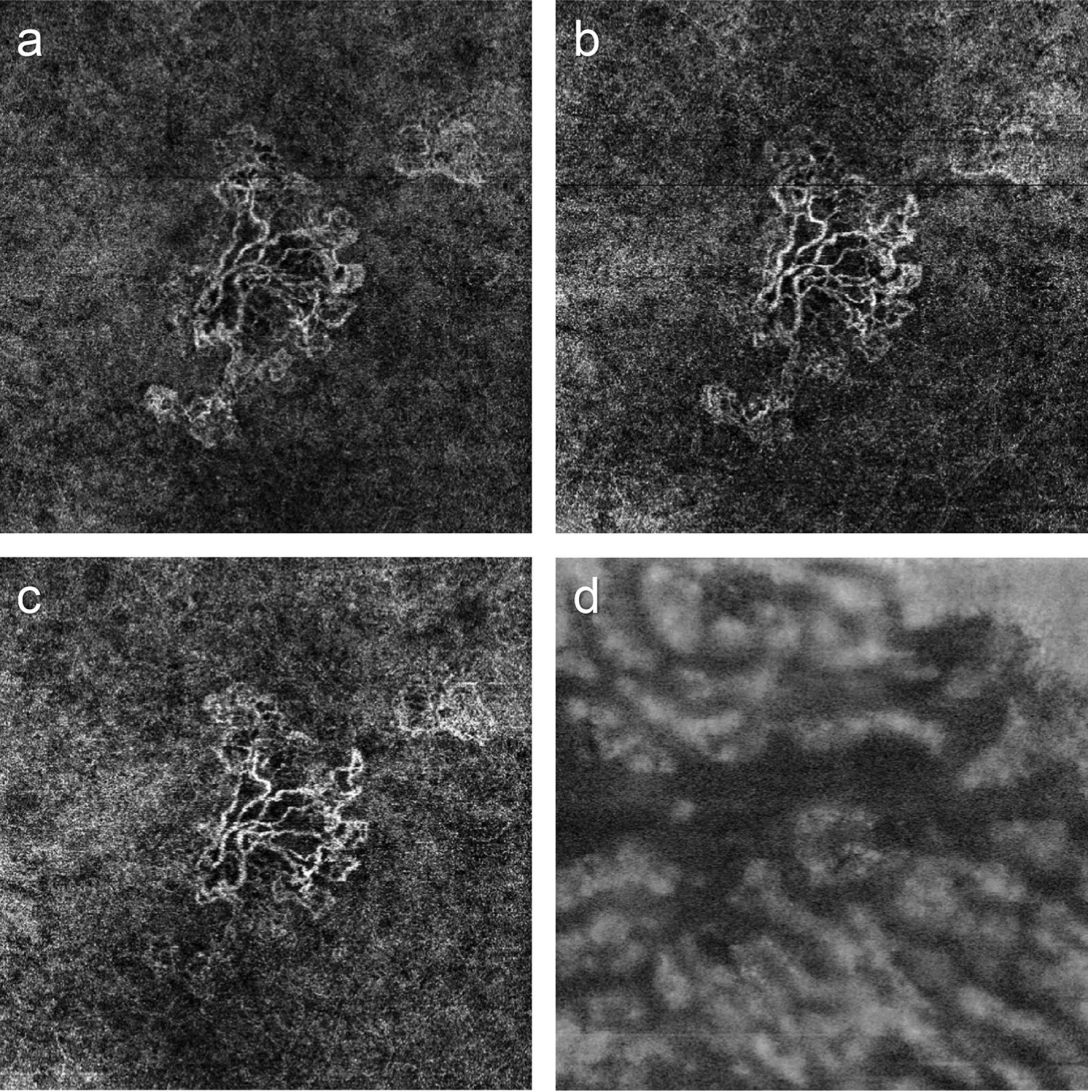
Changes in macular neovascularization (MNV) and Haller vessel morphology in eyes with no visual acuity gain and frequent injections. (**a**) Initial optical coherence tomography angiography image of a patient before the first injection shows MNV. At the initial visit, the best-corrected visual acuity was 20/40, which changed to 20/50 after 1 year. The total number of injections administered was 6. Central branches with peripheral anastomotic changes were present. The vessel area was 2.0 mm^2^, while the mean and maximal vessel diameters were 21.5 and 78.6 μm, respectively. (**b**) The central vessels were dilated after three loading injections, while peripheral anastomoses were not markedly shrunken. The vessel area was 2.2 mm^2^, while the mean and maximal vessel diameters were 20.7 and 65.5 μm, respectively. (**c**) After 1 year, the inferior part of the MNV had decreased, but the central vessel and peripheral anastomotic lesions retained their initial morphologies. The vessel area was 2.5 mm^2^, and the mean and maximal vessel diameters were 20.4 and 65.0 μm, respectively. (**d**) En face Haller vessel imaging after three loading doses showed dilation (the mean and maximum diameters were 144.3 and 851.5 μm, respectively) with a relatively shorter total length (41.1 mm).

**Figure 2:**
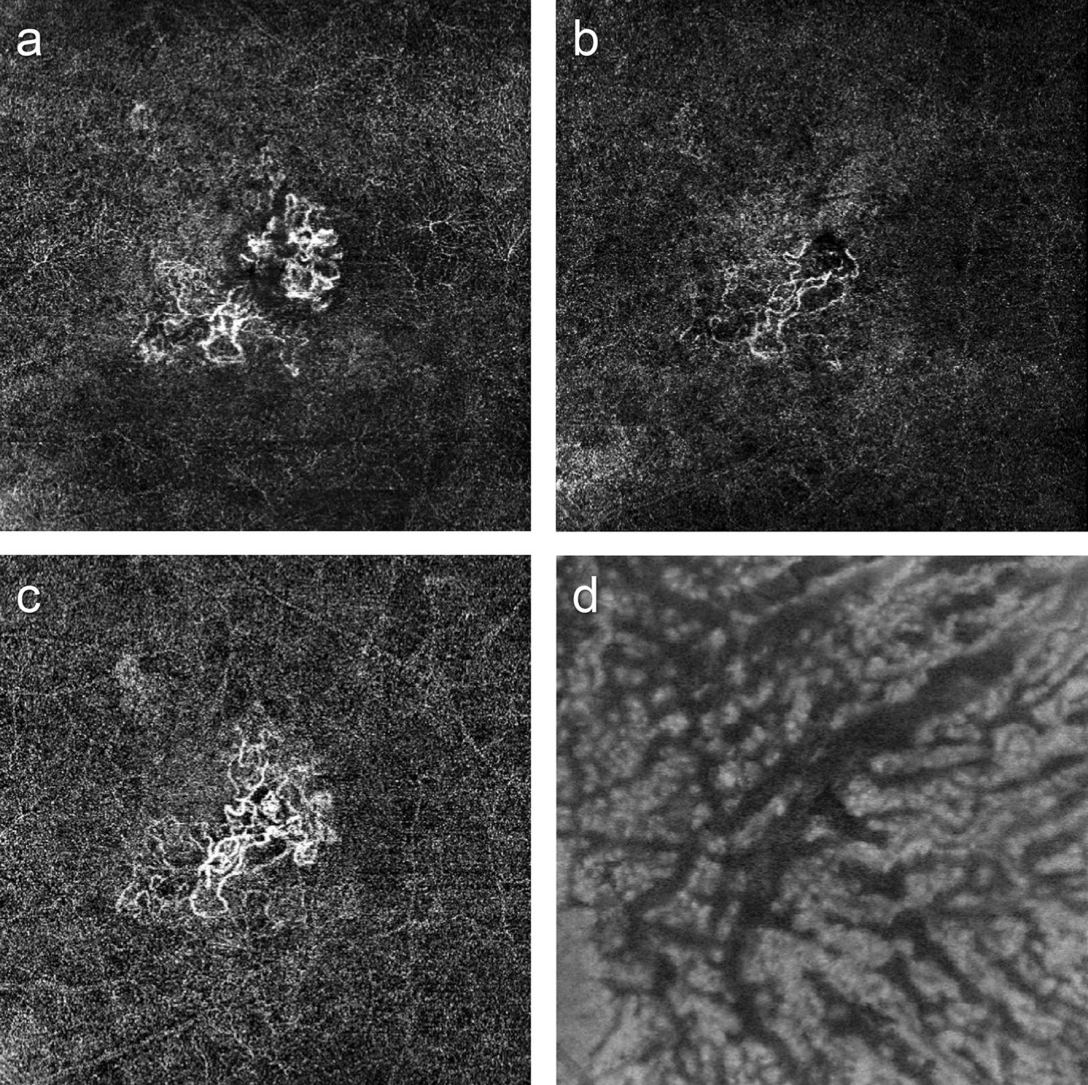
Changes in macular neovascularization (MNV) and Haller vessel morphology in eyes with visual acuity gain and infrequent injections. (**a**) Initial optical coherence tomography angiography image of a patient before the first injection shows MNV with well-developed peripheral anastomoses. The best-corrected visual acuity was 20/200 at the initial visit and 20/25 after 1 year. The total number of injections was 4. The vessel area was 1.1 mm^2^, while the mean and maximal vessel diameters were 22.2 and 65.5 μm, respectively. (**b**) After three loading injections, the vessel diameter decreased, as did the MNV size. The vessel area was 0.7 mm^2^, while the mean and maximal vessel diameters were 19.3 and 52.4 μm, respectively. (**c**) After 1 year, MNV and its diameter increased, but the overall morphology was not remarkably different from that of the initial MNV. The vessel area was 1.3 mm^2^, and the mean and maximal vessel diameters were 20.4 and 91.7 μm, respectively. (**d**) En face Haller vessel imaging after three loading doses showed dilation (diameter mean: 103.7 μm, diameter maximum: 497.8 μm) and a relatively longer total length (58.8 mm).

On univariate logistic regression analysis for each morphological factor adjusted for age and sex, infrequent group was associated with a smaller median and SD of Haller vessel diameter after three loading doses and with longer mean total Haller vessels after three loading doses and at 12 months (Table 1, Figs. 1, 2, Supplementary Figs. 1, 2). No MNV-associated factors were found to be significant (Table 1). Multivariate regression analysis revealed that the infrequent group had longer total Haller vessels after three loading doses (odds ratio = 3.05, P = 0.01).

**Table 1:** Univariate logistic regression analysis of the infrequent injection group based on en face Haller vessel and MNV parameters.

Parameters	En face Haller vessel						Parameters	MNV											
	After loading			At 12 months				Baseline			After loading			At 12 months			Baseline—after loading		
	OR	95% CI	P	OR	95% CI	P		OR	95% CI	P	OR	95% CI	P	OR	95% CI	P	OR	95% CI	P
**Diameter**							**Diameter**												
Mean	0.64	0.37–1.11	0.11	0.75	0.40–1.44	0.39	Mean	0.19	0.01–6.18	0.35	0.06	0.00–2.73	0.15	0.14	0.00–86.03	0.55	2.62	0.08–88.22	0.59
SD	0.48	0.25–0.92	0.03	0.64	0.30–1.34	0.23	SD	0.14	0.00–6.63	0.32	0.06	0.00–1.57	0.09	0.22	0.00–74.91	0.61	5.07	0.20–127.12	0.32
Median	0.60	0.36–0.99	0.04	0.68	0.38–1.24	0.21	Median	0.49	0.14–1.75	0.27	0.56	0.20–1.59	0.28	0.37	0.07–2.04	0.25	1.07	0.44–2.60	0.88
Maximum	0.97	0.90–1.04	0.41	0.91	0.80–1.03	0.12	Maximum	0.85	0.52–1.38	0.50	0.71	0.41–1.24	0.23	1.05	0.60–1.81	0.87	1.11	0.68–1.80	0.68
Total vessel length	3.13	1.31–7.49	0.01	13.84	1.84–104.00	0.01	Total vessel length	0.99	0.87–1.13	0.91	0.98	0.83–1.14	0.76	0.97	0.85–1.11	0.66	1.00	1.00–1.00	0.63
No. of intersection	1.02	1.00–1.03	0.06	1.02	1.00–1.04	0.07	No. of intersection	1.00	1.00–1.00	0.98	1.00	1.00–1.00	0.77	1.00	1.00–1.00	0.80	1.00	1.00–1.00	0.52
**Branch vessel length**							**Branch vessel length**												
Mean	0.89	0.69–1.16	0.41	0.85	0.57–1.27	0.43	Mean	0.18	0.03–1.03	0.05	0.94	0.83–1.08	0.40	2.31	0.27–19.46	0.44	0.49	0.14–1.69	0.26
SD	0.88	0.73–1.06	0.17	0.90	0.67–1.21	0.47	SD	0.26	0.07–1.01	0.05	0.86	0.42–1.77	0.68	0.97	0.31–2.98	0.96	0.67	0.30–1.51	0.33
FD	3618.52	0.00–1.17 × 10^12^	0.41	2.73 × 10^10^	0.01–1.23 × 10^23^	0.11	FD	1.61	0.01–180.52	0.84	2.99	0.03–294.19	0.64	0.10	0.00–474.13	0.60	0.17	0.00–321.62	0.64
Lacunarity	0.14	0.00–77.30	0.54	0.00	0.00–150.48	0.31	Lacunarity	3.45	0.06–201.07	0.55	2.03	0.01–705.95	0.81	389.50	0.02–8.57 × 10^6^	0.24	3.82	0.03–546.06	0.60
Vessel area	1.22	0.08–19.01	0.89	3.42	0.10–113.90	0.49	MNV area	0.88	0.27–2.89	0.83	0.79	0.22–2.75	0.71	0.79	0.22–2.79	0.71	10.93	0.03–4616.92	0.44
SFCT	1.00	0.99–1.00	0.30	1.00	0.99–1.00	0.45	Vessel area	0.76	0.02–32.05	0.89	0.40	0.00–38.06	0.69	0.61	0.01–27.02	0.80	127.63	0.00–9.60 × 10^7^	0.48

Age and sex were adjusted. Unit of vessel area, mm^2^; unit of total vessel length, mm; unit of diameter and branch vessel length, 10 μm.

MNV, macular neovascularization; Baseline—after loading, baseline value of parameter subtracted by the value after loading doses; No., number; SD, standard deviation; FD, fractal dimension, SFCT, subfoveal choroidal thickness; OR, odds ratio; CI, confidence interval.

### Haller vessel and MNV parameters associated with visual gain

Univariate logistic regression for each morphological factor adjusted by age, sex, and baseline visual acuity revealed that visual gain group was associated with a smaller maximal MNV diameter after three loading doses and a longer mean MNV branch length at 12 months. Additionally, the visual gain group was associated with a larger decrease in the maximal MNV diameter and the MNV area after three loading doses than at baseline (Table 2, Figs. 1, 2, Supplementary Figs. 1, 2). No Haller vessel-associated factors were found (Table 2). Multivariate regression analysis revealed that visual gain was associated with smaller maximal MNV diameters after three loading doses (odds ratio = 0.22, P = 0.03).

**Table 2:** Univariate logistic regression analysis of visual acuity gain group based on Haller vessel and MNV parameters.

Parameters	Haller vessel						Parameters	MNV											
	After loading			At 12 months				Baseline			After loading			At 12 months			Baseline—after loading		
	OR	95% CI	P	OR	95% CI	P		OR	95% CI	P	OR	95% CI	P	OR	95% CI	P	OR	95% CI	P
**Diameter**							**Diameter**												
Mean	0.91	0.42–1.97	0.82	1.42	0.64–3.16	0.39	Mean	1.36	0.01–181.95	0.90	0.80	0.01–108.59	0.93	974.79	0.13–7.32 × 10^6^	0.13	1.69	0.01–224.08	0.83
SD	0.78	0.37–1.63	0.51	1.01	0.41–2.45	0.99	SD	3.73	0.01–1323.81	0.66	0.20	0.00–27.90	0.53	190.76	0.03–1.07 × 10^6^	0.23	23.07	0.07–7488.65	0.29
Median	0.91	0.46–1.81	0.79	1.35	0.63–2.87	0.44	Median	2.78 × 10^6^	0.00-	1.00	0.84	0.19–3.85	0.83	2.01	0.20–20.02	0.55	1.95	0.48–7.81	0.35
Maximum	0.93	0.80–1.08	0.32	0.98	0.86–1.12	0.76	Maximum	1.01	0.43–2.35	0.99	0.31	0.11–0.88	0.03	0.63	0.24–1.68	0.36	4.31	1.24–14.99	0.02
Total vessel length	3.44	0.58–20.42	0.17	8.27	0.90–75.77	0.06	Total vessel length	0.90	0.73–1.11	0.34	0.64	0.28–1.45	0.28	0.82	0.60–1.11	0.19	1.00	1.00–1.00	0.44
No. of intersection	1.01	0.98–1.03	0.69	1.03	0.99–1.06	0.10	No. of intersection	1.00	1.00–1.00	0.34	0.99	0.98–1.00	0.19	1.00	0.99–1.00	0.26	1.00	1.00–1.01	0.49
**Branch vessel length**							**Branch vessel length**												
Mean	0.99	0.66–1.49	0.96	0.90	0.55–1.48	0.68	Mean	3.67	0.29–46.29	0.31	0.97	0.81–1.17	0.79	939.50	1.41–6.25 × 10^5^	0.04	3.79	0.42–34.14	0.23
SD	1.06	0.82–1.36	0.67	0.90	0.62–1.31	0.58	SD	4.87	0.50–47.72	0.17	1.38	0.48–4.00	0.55	10.63	1.03–109.53	0.05	1.14	0.34–3.86	0.83
FD	1.14	0.00–7.06 × 10^12^	0.99	7.34 × 10^7^	0.00–2.64 × 10^23^	0.32	FD	0.82	0.00–1427.09	0.96	0.27	0.00–262.50	0.71	229.46	0.00–2.22 × 10^8^	0.44	11.62	0.00–3.28 × 10^5^	0.64
Lacunarity	0.01	0.00–515.44	0.39	0.00	0.00–122.90	0.19	Lacunarity	1.68	0.01–507.64	0.86	0.17	0.00–2655.38	0.72	0.02	0.00–8237.32	0.56	6.54	0.01–7962.16	0.60
Vessel area	2.52	0.03–197.45	0.68	40.84	0.20–8510.32	0.17	MNV area	0.40	0.06–2.56	0.34	0.07	0.00–15.04	0.33	0.16	0.01–2.57	0.20	3.55 × 10^7^	4.69–2.68 × 10^14^	0.03
SFCT	1.00	0.99–1.01	0.89	1.00	0.99–1.01	0.88	Vessel area	0.05	0.00–22.27	0.34	0.00	0.00–57,144.15	0.27	0.00	0.00–49.28	0.23	1.21 × 10^5^	0.00–1.87 × 10^14^	0.28

Age, sex, and baseline visual acuity were adjusted. Unit of vessel area, mm^2^; unit of total vessel length, mm; unit of diameter and branch vessel length, 10 μm.

MNV, macular neovascularization; Baseline—after loading, baseline value of parameter subtracted by the value after loading doses; No., number; SD, standard deviation; FD, fractal dimension, SFCT, subfoveal choroidal thickness; OR, odds ratio; CI, confidence interval.

### ROC analysis of the injection frequency and visual gain

**Figure 3:**
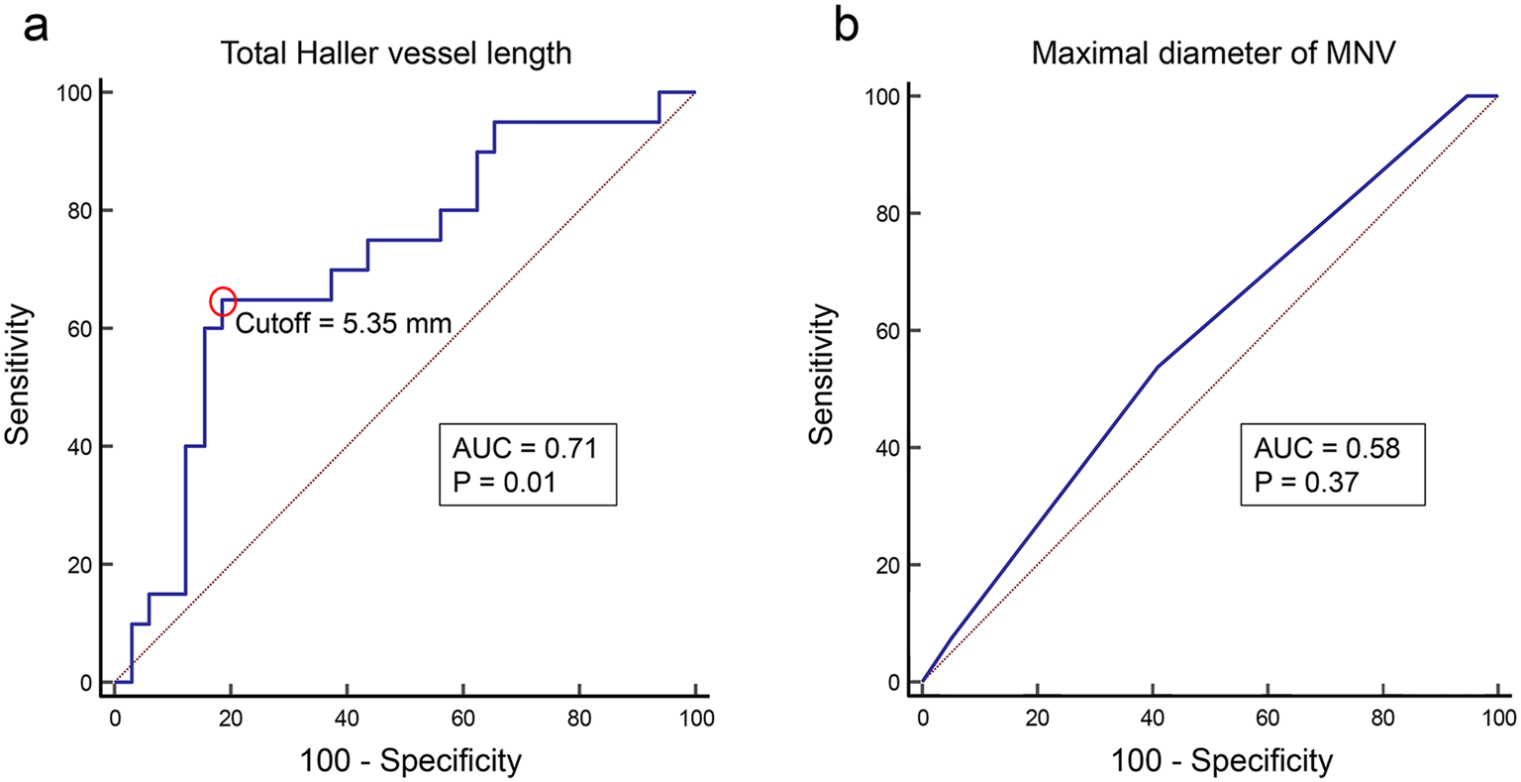
The receiver operating characteristic (ROC) curve based on statistically significant factors identified using multiple logistic regression analyses for visual acuity gain and number of treatments over 1 year. (**a**) ROC curve of the injection frequency based on the total Haller vessel length; the area under the curve (AUC) was as high as 0.71 (P = 0.01). The cutoff value was set at 5.35 mm (open red circle). (**b**) ROC curve of the visual acuity gain based on the maximal macular neovascularization diameter. The AUC was 0.58, although no statistical significance was observed (P = 0.37).

The ROC curve to predict the injection frequency cutoff based on the total length of the Haller vessel after three loading doses is shown in Fig. 3a. The area under the curve (AUC) was 0.71 (P = 0.01) with a sensitivity of 65.0%, specificity of 81.2%, positive predictive value of 68.4%, and negative predictive value of 78.8% when the cutoff value was set at 5.35 mm. The ROC curve to predict the visual gain based on the maximal MNV diameter after three loading doses is shown in Fig. 3b. The AUC was 0.58 (P = 0.37), with a sensitivity of 53.9%, specificity of 59.0%, positive predictive value of 30.4%, and negative predictive value of 79.3%.

## Discussion

Our study identified early-phase parameters that can predict the required number of anti-VEGF injections and visual changes in treatment-naïve active nAMD using longitudinal images of the MNV and en face Haller vessel images at baseline, after three loading doses, and at 12 months. Previous investigators identified several OCTA markers in nAMD patients undergoing IVI treatments^3,4,6,14,15^. Moreover, the longitudinal changes in Haller vessel morphology on en face imaging combined with MNV changes have not been addressed thoroughly.

In this study, the diameter and total length of the Haller vessel were significantly associated with injection frequency. On univariate logistic regression, infrequent injection group was associated with a smaller Haller vessel diameter (median and SD) and longer mean total Haller vessel length after three loading doses. A smaller diameter contrasts with the characteristics of pachyvessels, which were recently shown to be associated with the development of MNV^7,8^. According to Poiseuille’s rule regarding hemodynamics, shorter Haller vessels with larger diameters contribute to lower resistance, thereby increasing blood flow^16^. This increased blood flow may provide sufficient volumes of oxygen and nutrition support to the pathological MNV tissue despite repetitive anti-VEGF treatments. Therefore, a smaller diameter and longer Haller vessel might lower the probability of maintaining MNV. Moreover, given the lower SD of the Haller vessel diameter associated with patients in the ‘infrequent’ group, maintaining a homogeneously smaller vessel diameter might also be important for preventing overflowing. However, these results can also be interpreted oppositely. The lowered flow of Haller vessels could aggravate the ischemic environment, leading to an increase in VEGF levels and more active MNV. Therefore, the interpretation of smaller diameter and longer length of Haller vessel solely based on Poiseuille’s rule might not fully explain the lower injection frequency over 12 months. Interestingly, a recent study revealed that the foveal choroidal vessel diameter measured in OCT B-scan was reduced after loading doses in PCV, and this diameter increased at the time of recurrence, suggesting a positive relationship between the choroidal vessel diameter and MNV activity^17^. While this result was based on PCV eyes and the choroidal diameter was measured in OCT B-scan, this association is consistent with our results, in which eyes requiring a lower injection frequency had a smaller Haller vessel diameter observed in en face OCT image. Further study is needed to determine level of cytokines, including VEGF, in the vitreous or aqueous humor level according to the morphology of en face Haller vessel to clarify their relationship. Meanwhile, both univariate and multivariate regression analyses showed that infrequent injections were associated with a longer total Haller vessel length after three loading doses with a high AUC of 0.71. Hence, a longer total Haller vessel length in the early disease phase might be a more important predictor of outcome than a smaller mean diameter.

Conversely, the prognostic value of MNV related to injection frequency was not significant. The infrequent group showed a significantly shorter branch length (mean and SD) of MNV at baseline than the infrequent group. The shorter branch length, which might partially represent more angiogenic budding vessels, may be more likely to regress than it would in MNVs with longer branch lengths when administered the same anti-VEGF dose. However, logistic regression analysis showed that the MNV parameters were not associated with injection frequency. Likewise, the vessel diameter (mean, median, and SD) of MNV was not associated with injection frequency. We expected that the greater the vessel thickness, the more mature the vessels would be, leading to a greater resistance to anti-VEGF treatment, requiring more injections^18^. This unexpected result might be explained by the simplified morphology of the binarized MNV during imaging, which may have led to an underestimation of the detailed microvascular changes.

Visual acuity gain over 12 months was highly associated with early MNV parameters but not with Haller vessel characteristics. On univariate logistic regression analysis, visual acuity gain was associated with a smaller maximal diameter of MNV and a larger decrease in this maximal diameter of MNV after three loading doses. Multivariate regression also showed the significance of a smaller maximal diameter of MNV after three loading doses. The MNV diameter attracted attention after Spaide suggested the involvement of arteriogenesis^18^. Repetitive anti-VEGF treatments induce the closure of peripheral capillaries and lead to dilation of preexisting channels by remodeling the vessel wall via proinflammatory signals, while smooth muscle cells contribute to the resistance to VEGF withdrawal^18^. However, in the early disease phase of treatment-naïve patients such as ours, the vessel diameter might be less affected by arteriogenesis and may be dominated by innate angiogenesis. Therefore, a greater reduction in the maximal MNV diameter after three loading doses might represent greater sensitivity to anti-VEGF agent, which eventually translates to visual acuity gain. Meanwhile, the AUC for visual gain based on the maximal MNV diameter was low and lacked significance (AUC = 0.58, P = 0.37). Therefore, further investigations with a larger population might be needed to clarify the implication of MNV diameter-related parameters.

There might be a concern that the different characteristics of en face Haller vessel morphology in each subgroup originate from the different ratio of PCV included in the subgroup, which conveys larger diameter of Haller vessels. The ratio of PCV to typical nAMD and SFCT were not different between subgroups classified by injection frequencies (Supplementary Table 1). Also, the ratio of PCV and SFCT were not different between subgroups classified by visual gain (Supplementary Table 1). Therefore, difference in Haller vessel characteristics between subgroups might have not originated from the different ratio of PCV to typical nAMD, or the different distribution of SFCT between subgroups.

Our study has several limitations. First, we could not compare the Haller vessel morphology at baseline and after three loading doses, as we did with MNV, because an adequate en face image of the Haller vessel could be obtained only after three loading doses and after 12 months. At baseline, the combination of fluid, hemorrhage and MNV area induced blurring and improper segmentation of the underlying Haller vessel. Improving imaging technologies may better visualize vascular changes in the Haller vessel. Second, images gathered at 12 months were smaller, and direct comparison against either baseline or after three loading doses could not be performed due to the omission of the required images. A prospective study design might overcome this drawback, which is characteristic of a retrospective study design. Third, Haller vessel and MNV images may not show intact morphologies due to layer segmentation errors. The development of improved segmentation algorithms using advanced techniques such as deep learning might overcome this limitation.

In conclusion, our study revealed that quantitative parameters obtained from en face imaging of the Haller vessel and MNV could predict the required injection frequency and visual outcome in treatment-naïve nAMD.

## Methods

### Participants

We performed a retrospective review of patients with nAMD who visited the Konkuk University Medical Center between October 2017 and December 2020. This study followed the tenets of the Declaration of Helsinki and was approved by the Institutional Review Board of the Konkuk University Medical Center (KUMC 2021-04-063); the Board waived the requirement for written informed consent because of the study’s retrospective design.

The inclusion criteria were age 50 years or older, treatment-naïve nAMD confirmed by multimodal retinal imaging (fluorescein angiography, indocyanine green angiography, and/or OCT), and at least 12 months of follow-up data. After three monthly injections with ranibizumab or aflibercept loading doses, further injections were performed based on the pro re nata regimen, which includes monthly observations with treatment resumption based on disease activity (e.g., intraretinal/subretinal fluid or hemorrhage) seen on OCT^19^. The exclusion criteria were a history of high myopia (< − 6 diopters), images obscured by media opacity, significant cataract progression without surgery during the follow-up period that affected image quality or caused visual loss, fibrotic scarring, atrophic changes at the fovea, and/or any concurrent progressive retinal disease or other combined retinal diseases. We also excluded poor OCTA images with artifacts. Among the en face structural OCT images of the choroidal layer, overly dark images filled with extremely thick outer choroidal vessels were excluded as it was not possible to detect the proper central vessel line required to generate en face Haller images.

Among nAMD, typical nAMD was defined as nAMD containing type 1 or 2 MNV confirmed by multimodal retinal imaging (fluorescein angiography, indocyanine green angiography [ICGA], and OCT). The diagnosis of PCV was based on the observation of elevated orange-red lesions on fundus examination and polypoidal lesions on ICGA.

During every visit, the best-corrected visual acuity assessment, slit-lamp examination, and dilated funduscopic examination were performed. OCTA images were obtained from all patients at the following three time points: at baseline before the first injection, 1 month after three loading doses, and 12 months after the baseline measurement. Structural en face OCT images were acquired at only two time points—after three loading doses and after 12 months—due to the presence of artifacts on Haller vessel images (i.e., blurring and distortion of the vessel image from intraretinal/subretinal fluid and/or dark shadows from subretinal exudation or subretinal hemorrhage) that were found in a majority of the eye images (39 of 52) at baseline (Supplementary Fig. 3). At month 12, OCTA and en face structural images were acquired from 34 eyes (65.4%); medical records, including visual acuity and the number of injections, were also collected. At each visit, SFCT was measured using the built-in caliper tool in the OCT viewer.

### Acquisition of OCTA and en face OCT

OCTA images were acquired using a Spectralis OCT angiography (Spectralis HRA + OCT2^®^; Heidelberg Engineering, Heidelberg, Germany), and the scanned area was 6 × 6 mm. To obtain en face images of MNV, two expert retina specialists (H.L. and H.C.K.) adjusted the upper and lower borders of the preset choriocapillaris slab to identify the MNV plane and optimize the entire morphology of the MNV, as previously described^20^. Disagreement between the two retina specialists regarding the optimal position of the slab for MNV visualization was resolved by open discussion.

En face images of the Haller layer were obtained by adjusting the thickness of the slab to 10 µm and positioning it so that it spanned the center of the Haller layer in B scans to the greatest extent possible, as described in a previous study^11^. When the contour of the slab did not span the entire central line of all vessels in the Haller layer, the layer at the macula was prioritized. To validate this method, two retinal specialists (H.L. and H.C.K.) independently determined the slab for the en face Haller image, and the intergrader reliability was high enough to represent the suggested method to generate consistent en face Haller images among clinicians (Supplementary Fig. 4). Disagreement between the two retina specialists regarding the optimal position of the slab for en face Haller vessel visualization was resolved by open discussion. measurements were manually measured using Spectralis software.

### Quantitative evaluation of the Haller vessel using en face OCT and of MNV using OCTA

**Figure 4:**
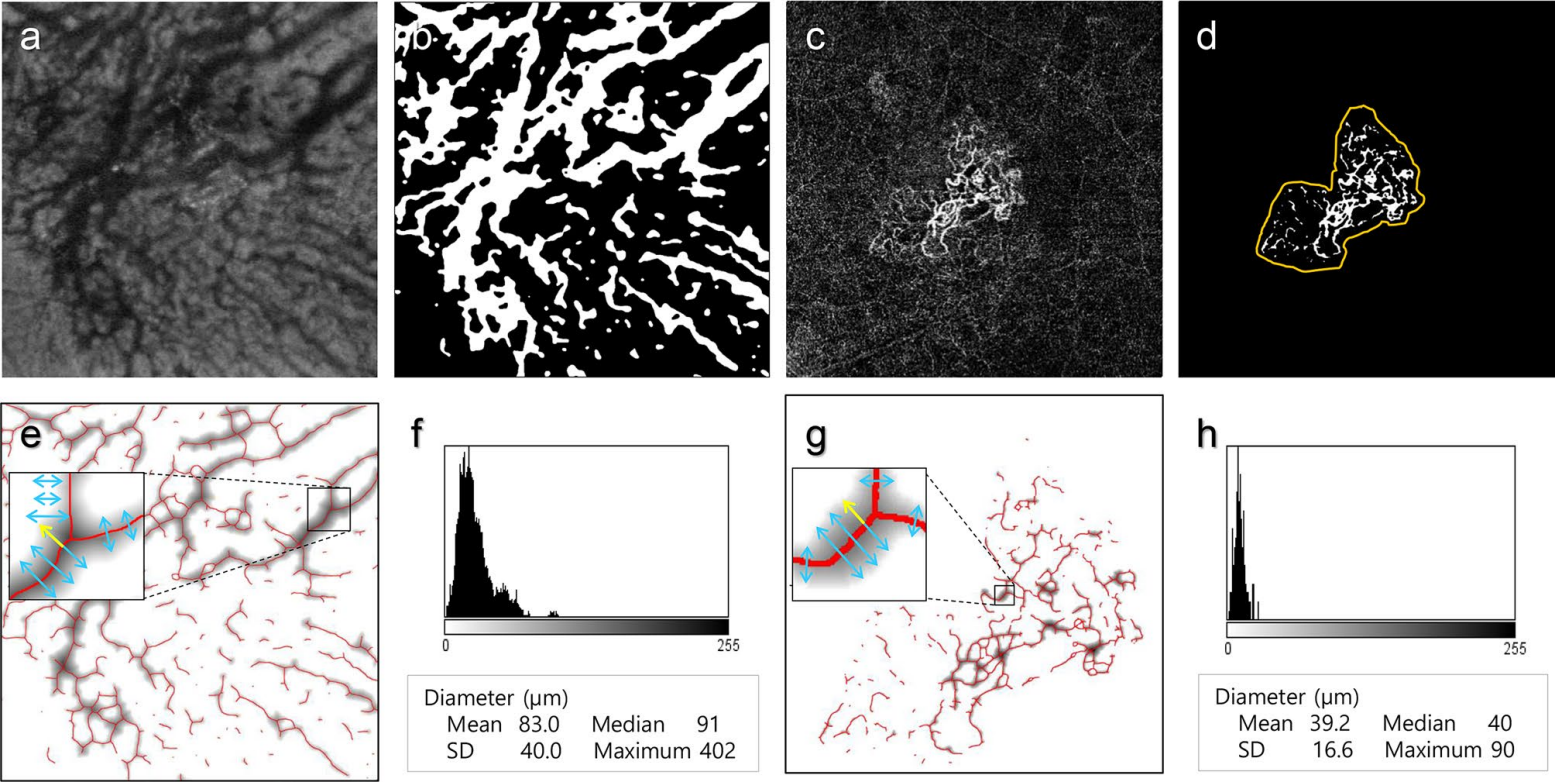
Quantitative measurement of various vessel-related parameters. (**a**) En face image of the Haller vessel structure in an eye with age-related macular degeneration. (**b**) Binarized en face image of a Haller vessel obtained via the Phansalkar binarization method using FIJI software. The vessel area was measured by counting white pixels. (**c**) En face morphology of macular neovascularization (MNV) was observed using optical coherence tomography angiography in the same eye. (**d**) Binarized image of MNV obtained using Weka binarization. The MNV area was calculated from the area inside the MNV boundary (orange-colored line), while the vessel area was measured by counting white pixels inside the MNV boundary. (**e**) The diameter of the en face Haller vessel was calculated using the DiameterJ plugin in FIJI. When the binarized vessel image of (**b**) is loaded, the DiameterJ plugin measures the distance between the vessel border (i.e., vessel diameter, blue arrows in inlet) by doubling the distance from every pixel of skeletonized vessel (red line in inlet) to the closest border of vessel (yellow arrow in inlet). The grayscale image along the skeletonized vessel represents the diameter of the vessel along the centerline of the vessel. The darkness of the gray-colored area along the centerline of the vessel denotes the distance to the nearest background pixel, which is proportional to the vessel diameter at that pixel point. (**f**) Histogram showing the distribution of diameters at each point on the centerline of the vessel. The X-axis is proportional to the diameter, and the Y-axis shows the frequency of each diameter. After repetitive measurement of vessel diameter at each pixel on the centerline of vessel, the sum of total diameter values are divided by the pixel numbers of the skeletonized vessel to calculate the mean diameter. Additionally, the median, maximum and standard deviation of vessel diameter can be calculated from these data, including multiple diameter values along the centerline of the vessel. (**g**) The diameter-associated parameters of the en face MNV were calculated using the same methods applied to the Haller vessel image, (**e**) and (**f**), using the DiameterJ plugin in FIJI. (**h**) Histogram showing the distribution of diameters at each point on skeletonized vessels of MNV. From these data, the mean, median, maximum and standard deviation of the MNV diameter can be calculated.

Phansalkar binarization (radius 100) was applied to binarize the Haller vessel from the en face OCT image using FIJI software (National Institutes of Health, Bethesda, MD) (Fig. 4a,b)^21^. The MNV could be optimally binarized by the Weka segmentation method in FIJI (Fig. 4c,d). The vessel areas of the Haller vessel and MNV were measured by counting all pixels of the binarized vessels (Fig. 4a–d). The MNV area was calculated from the area inside the MNV boundary (Fig. 4d. orange-colored line).

In both the en face Haller vessel and MNV, the vessel diameter is not uniform in a single image; therefore, the distribution of vessel diameter should be extracted to accurately evaluate the characteristics of vessel diameter as their mean, median, maximum and SD. These diameter-related parameters were calculated using the DiameterJ plugin for the en face Haller vessel (Fig. 4e,f) and MNV images (Fig. 4g,h)^22^. This plugin was validated for extracting information regarding fibers of varying diameters^22^. In the binarized vascular image, the DiameterJ plugin measured the distance between the vessel border (i.e., vessel diameter, blue arrows in inlet in Fig. 4e,g) at every pixel of the vessel centerline (red line in inlet in Fig. 4e,g) by finding the shortest distance from each pixel point to the background (yellow arrow in inlet in Fig. 4e,g). The sum of diameters from each pixel on the centerline was divided by the total number of pixels of the centerline to calculate the mean diameter of the vessel in an image (Fig. 4f,h). The median, maximum and standard deviation of diameters along the whole vessels in an image were also calculated from the distribution of the diameters (Fig. 4f,h).

After skeletonization of the binarized Haller vessels, intersections were counted using the ‘Analyze skeleton’ function in FIJI. Based on the skeletonized image, branch vessel lengths were obtained by measuring vessel lengths between intersections and vessel lengths between intersections and end points (Supplementary Fig. 5a). Additionally, the branch vessel lengths could be obtained by measuring the vessel lengths between different intersections and those between intersections and endpoints when ‘Analyze skeleton’ was applied. Furthermore, the total vessel length was calculated as the sum of all branch vessel lengths.

The FD and lacunarity of the vascular network were calculated based on the binarized Haller vessel and MNV image using the FracLac plugin to quantify the complexity of the Haller vessel^23^. The box-counting method was applied to estimate the FD and lacunarity of the vascular network. The box-counting method approximates the FD by covering the vessel image with a grid of boxes with a side length ε and counting the number of nonempty boxes as Nε. The box length ε is progressively reduced, and the corresponding number of nonempty boxes Nε is counted (Supplementary Fig. 5b,c)^24^. The slope of a log–log plot of Nε versus ε corresponds to the FD, and higher FD values represent a more complex pattern. Lacunarity is another metric representing complexity that describes the distribution of the gaps. Lacunarity is calculated from the coefficient of variation in pixel density according to the progressively reducing box^25^. For the MNV in the OCTA image, the same parameters, including intersection number, branch/total vessel lengths, FD and lacunarity, were calculated using the same method used for the analyses of en face Haller vessel images (Supplementary Fig. 5d–f).

### Grouping patients according to visual loss and treatment interval

Patients were grouped according to the number of IVIs of anti-VEGF drugs administered during the 12 months of follow-up. The ‘infrequent’ group required IVIs four or fewer times over 12 months, while the ‘frequent’ group required IVIs five or more times. Second, the subjects were divided into two groups according to visual gain (those whose visual acuity improved by logMAR 0.3 or more versus those with no such improvement).

### Statistical analysis

The Mann–Whitney test was performed to compare differences in the mean values, while the chi-square test was used to compare the ratios of men to women. A univariate logistic regression analysis was conducted for each parameter to assess quantitative parameters associated with clinical outcomes. The factors found to be significantly different between baseline and after three loading doses on univariate analysis (P < 0.05) were subjected to multivariate logistic regression analysis using backward stepwise selection. The intraclass correlation coefficient (ICC) was calculated to confirm the intergrader reliability of the slab position of OCTA to generate appropriate en face Haller vessel images. SPSS software version 18 (SPSS Inc., Chicago, IL, USA) was used for all statistical analyses. A P value of < 0.05 was considered significant.

The receiver operating characteristic (ROC) curve analysis of our constructed predictive model was performed using MedCalc version 19.0.6 (MedCalc Software, Ostend, Belgium).

## Supplementary Information

Supplementary Information.

## Data Availability

The datasets generated during and/or analyzed during the current study are not publicly available due to our hospital’s policy regarding patient records but are available from the corresponding author upon reasonable request.
