# Supplementary material for: Morphology of en face Haller vessel and macular neovascularization at baseline and 3 months as predictive factors in age-related macular degeneration
Source: Sci Rep. 2022 Jun 25;12:10821. doi: 10.1038/s41598-022-15139-0 (PMC9233682; doi:10.1038/s41598-022-15139-0)
Supplement: Supplementary file 1 — Supplementary Information. [file 41598_2022_15139_MOESM1_ESM.docx]

**Morphology of en face Haller vessel and macular neovascularization at baseline and 3 months as predictive factors in age-related macular degeneration**

Hyungwoo Lee, MD, PhD, SoHyeon Kim, MD, Myung Ae Kim, MD, Hyewon Chung, MD, PhD, Hyung Chan Kim, MD, PhD

Corresponding author: Hyung Chan Kim, MD, PhD, Department of Ophthalmology, Konkuk University Medical Center, 120-1 Neungdong-ro, Gwangjin-gu, Seoul, Republic of Korea, 05030; Phone: 82-2-2030-8198; Fax: 82-2-2030-5273; e-mail: eyekim@kuh.ac.kr

**Supplementary Fig. 1.** Changes in macular neovascularization (MNV) and Haller vessel morphology in eyes with no visual acuity gain and infrequent injections. (a) Initial optical coherence tomography angiography image of a patient before the first injection showed MNV with thicker central vessels and numerous tiny branches at the periphery. The best-corrected visual acuity on the initial visit was 20/25 and decreased to 20/40 after 1 year. The total number of injections was 3. The vessel area was 4.9 mm^2^, while the mean and maximal vessel diameters were 21.7 μm and 91.7 μm, respectively. (b) After three loading injections, the vessel diameters at the main branches were not significantly decreased, although the peripheral branches were slightly decreased. The vessel area was 4.3 mm^2^, while the mean and maximal vessel diameters were 19.6 μm and 78.6 μm, respectively. (c) After 1 year, the overall morphology of MNV was similar to that of baseline; the vessel area was 5.5 mm^2^, while the mean and maximal vessel diameters were 22.7 μm and 104.8 μm, respectively. (d) En face Haller vessel imaging after three loading doses showed dilation (diameter mean: 91.7 μm, diameter maximum: 484.7 μm) and a relatively longer total length (61.5 mm).


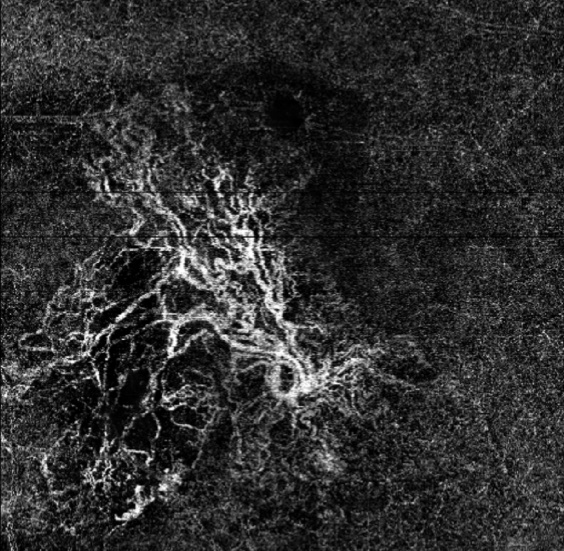

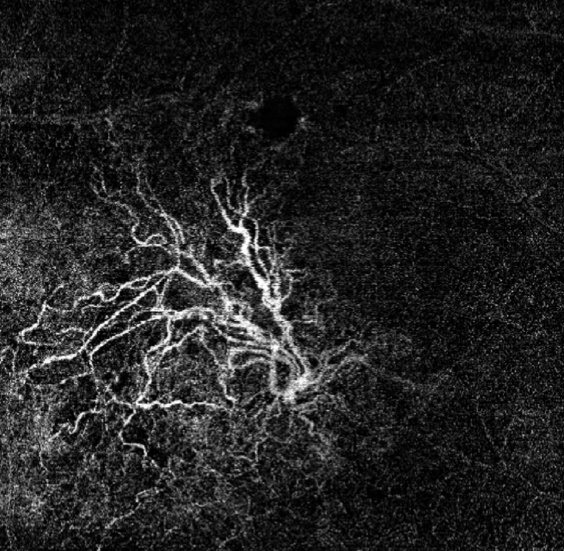

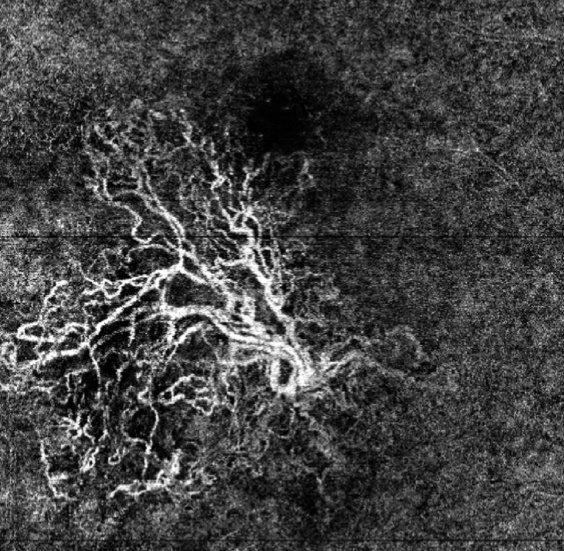

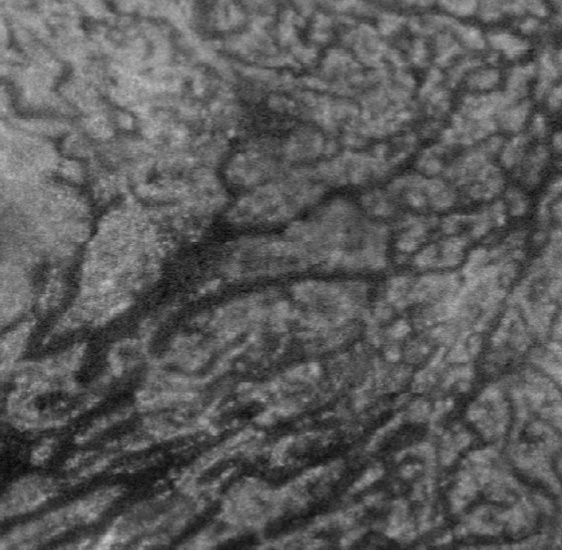


a

b

c

d

**Supplementary Fig. 2.** Changes in macular neovascularization (MNV) and Haller vessel morphology in eyes with visual acuity gain and frequent injections. (a) Initial optical coherence tomography angiography image of a patient before the first injection shows MNV without a defined central trunk or peripheral anastomosis. Note the thicker vessels at the central and inferior areas in MNV. The best-corrected visual acuity on the initial visit was 20/200 and improved to 20/60 after 1 year. The total number of injections was 6. The vessel area was 0.6 mm², and the mean and maximal vessel diameters were 19.1 μm and 52.4 μm, respectively. (b) After three loading injections, the vessel diameters in all the areas of MNV decreased. The vessel area was 0.4 mm², while the mean and maximal vessel diameters were 18.3 μm and 50.5 μm, respectively. (c) After 1 year, the vessel thickness was similar to that of the initial visit. The vessel area was 0.7 mm², with the mean and maximal vessel diameters being 21.0 μm and 65.5 μm, respectively. (d) En face Haller vessel imaging after three loading doses showed dilation (diameter mean: 101.2 μm, diameter maximum: 406.1 μm) and a relatively shorter total length (42.8 mm).


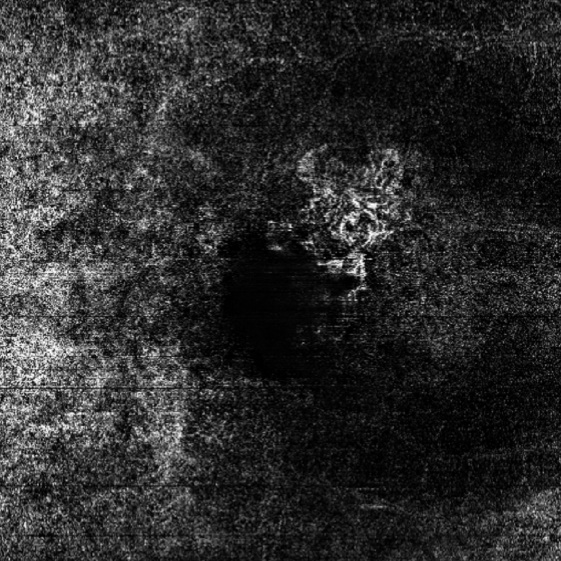

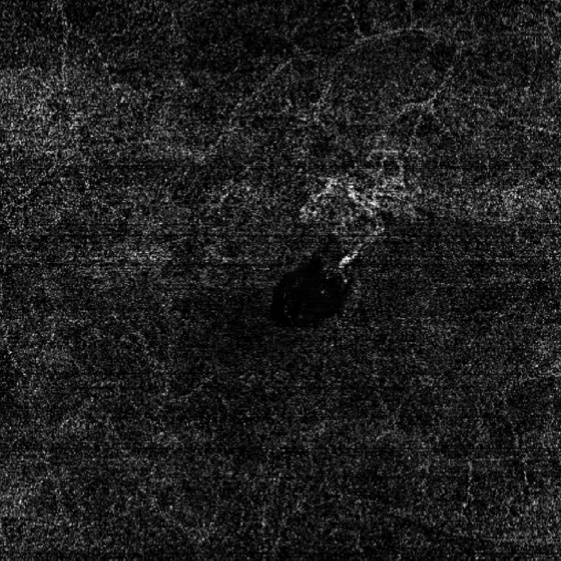

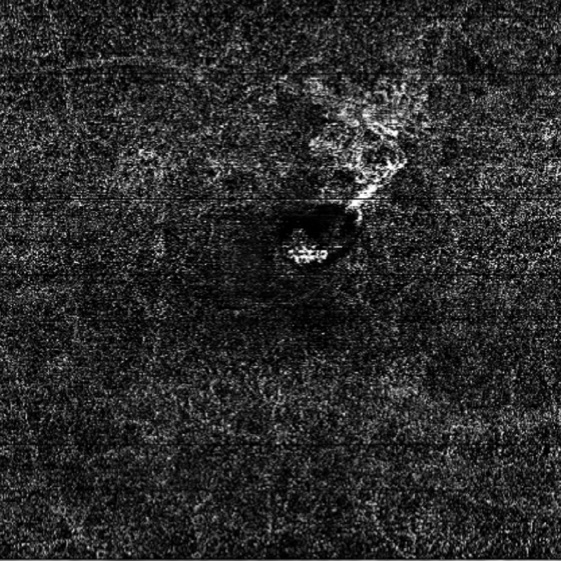

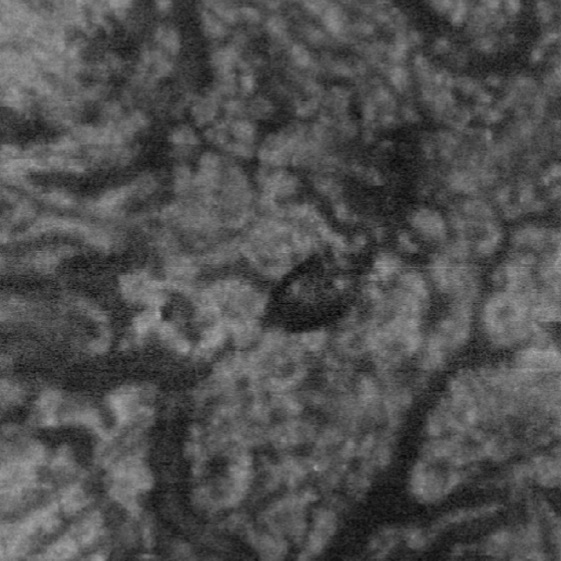


a

b

c

d


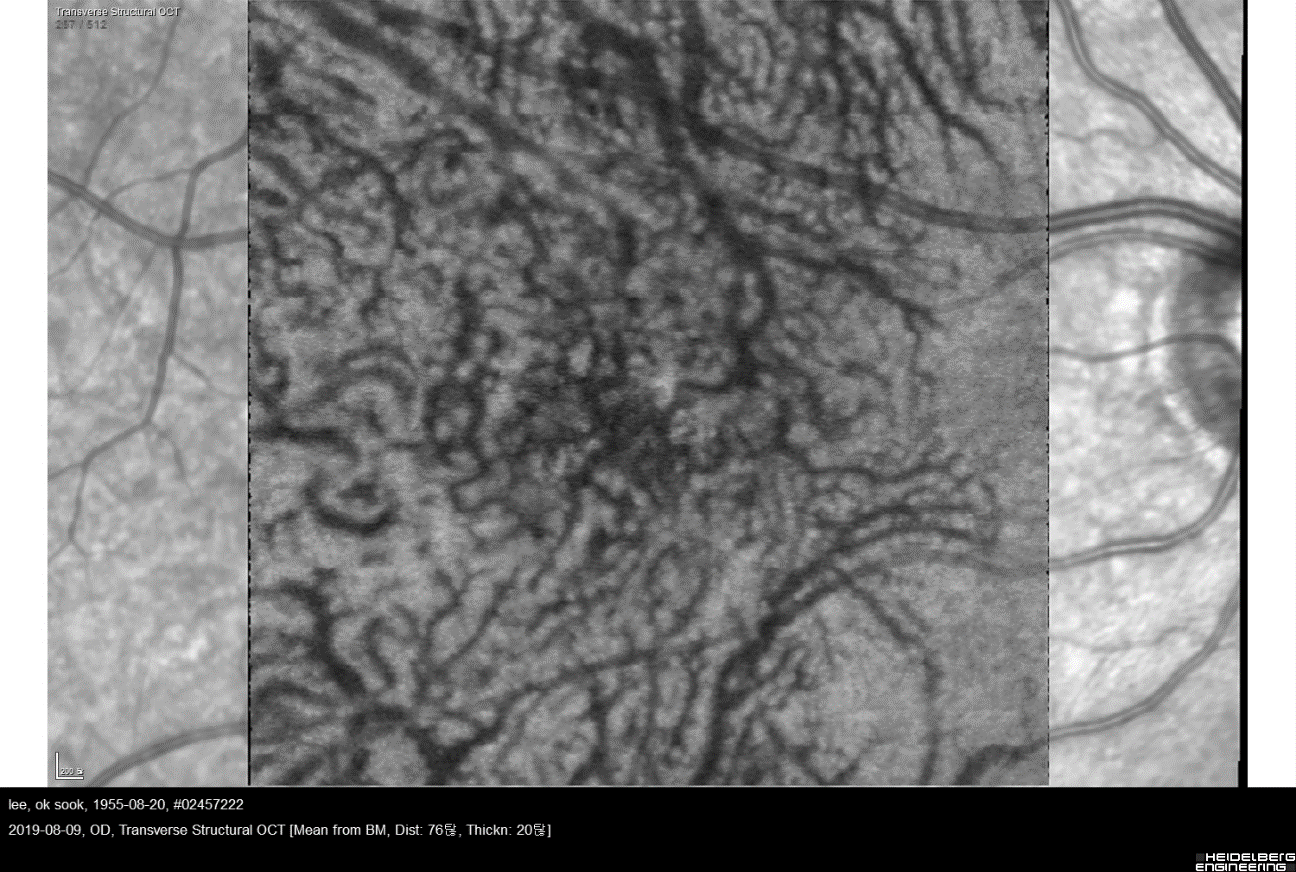

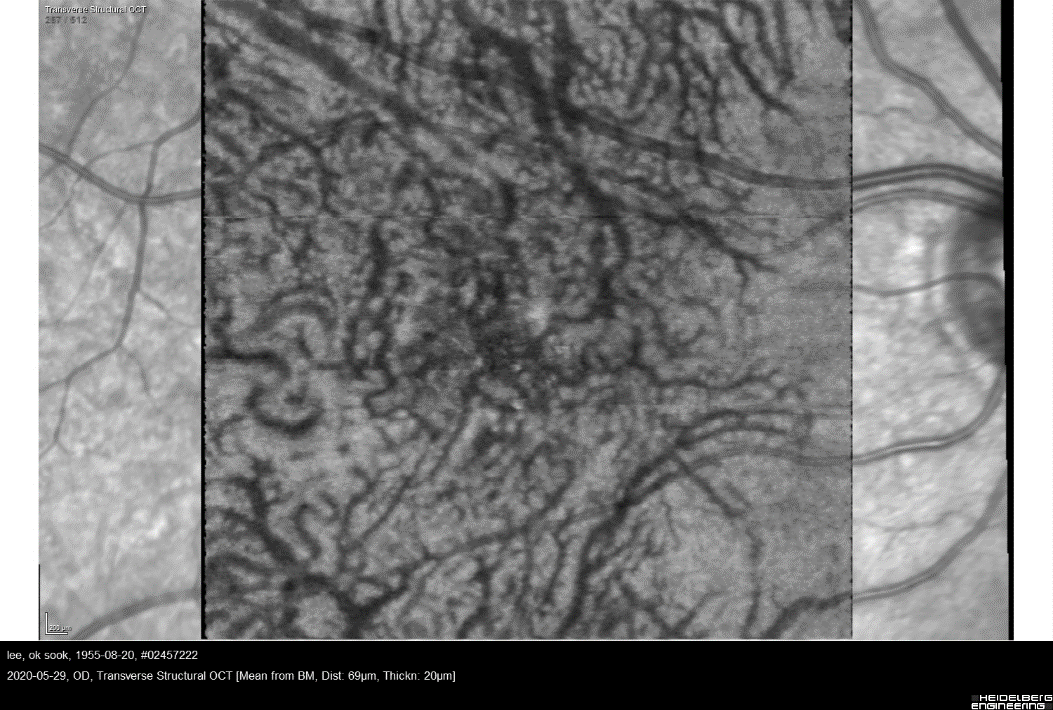

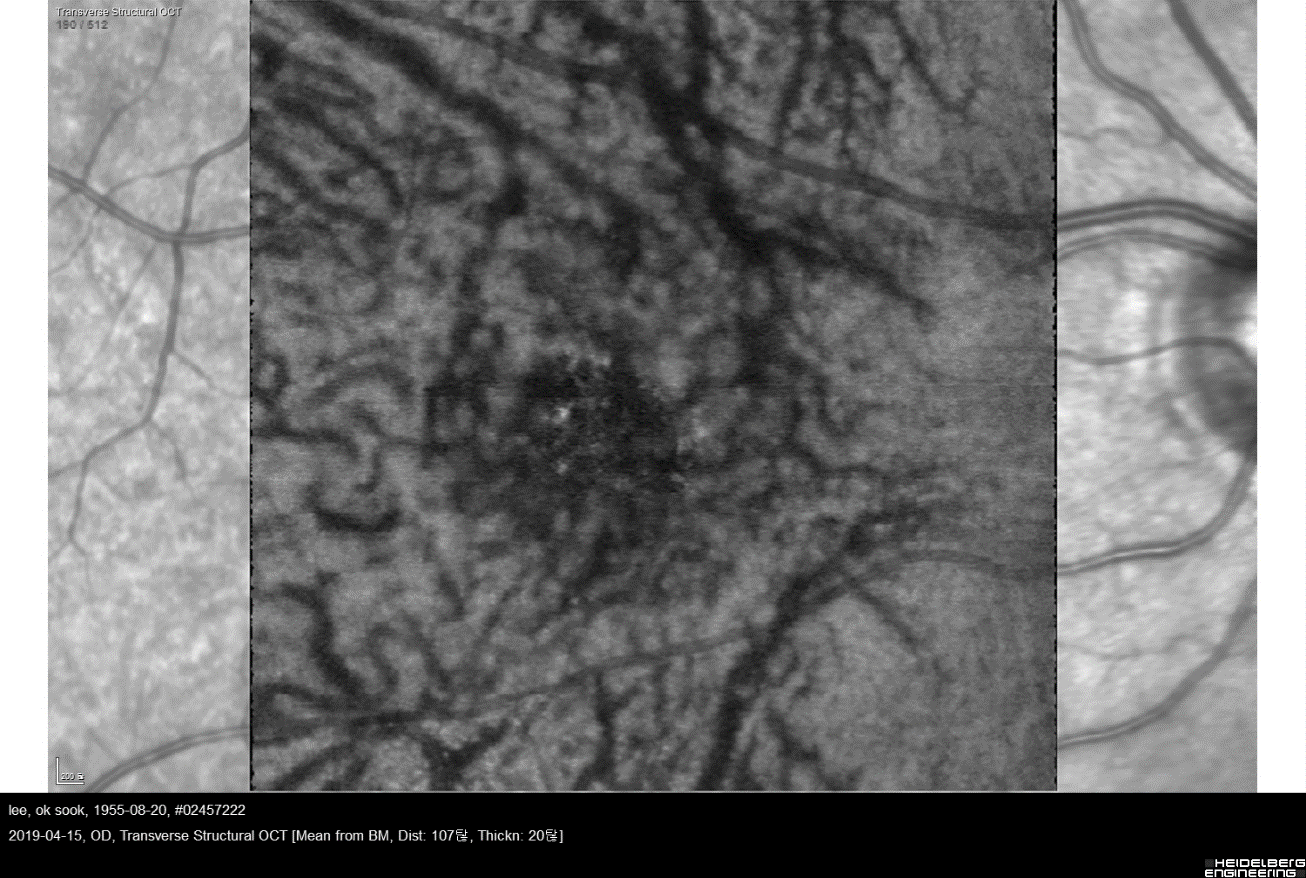

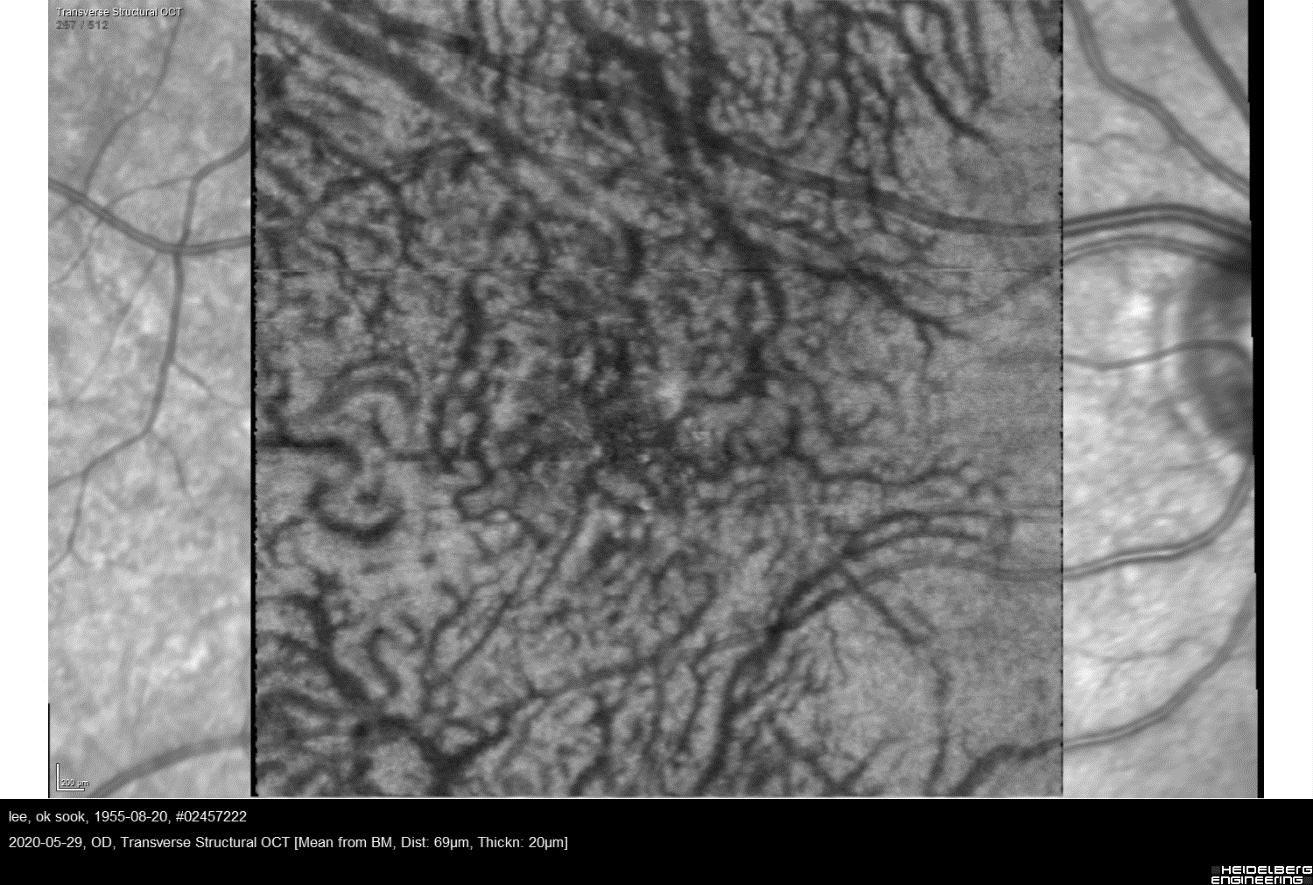

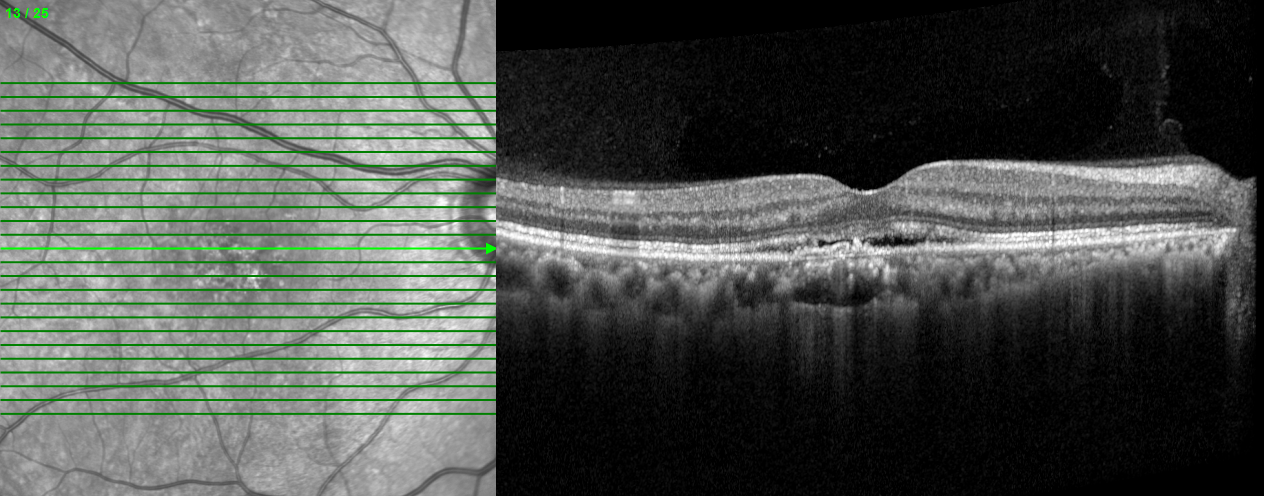

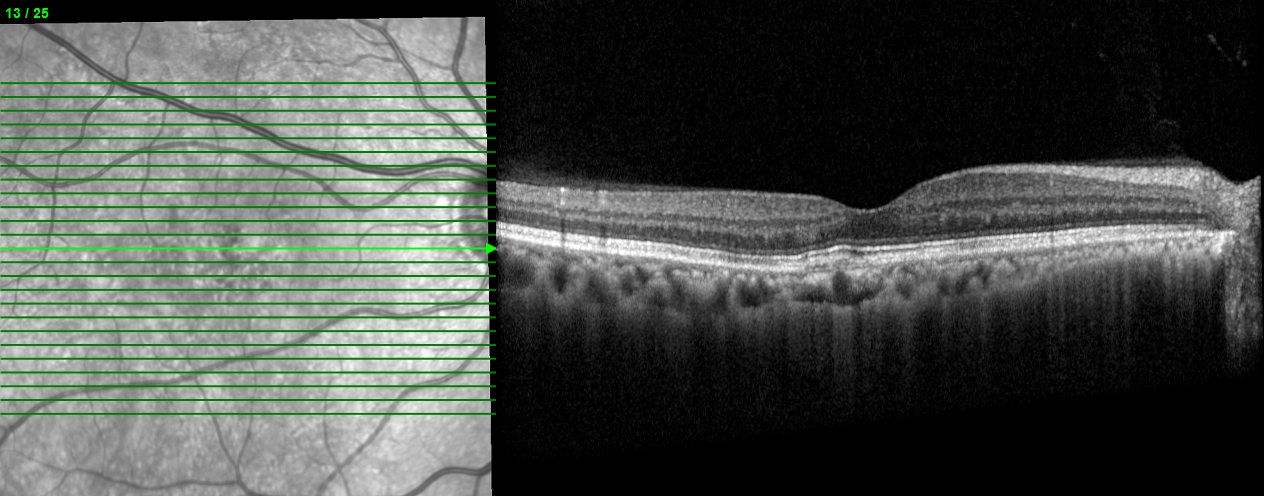

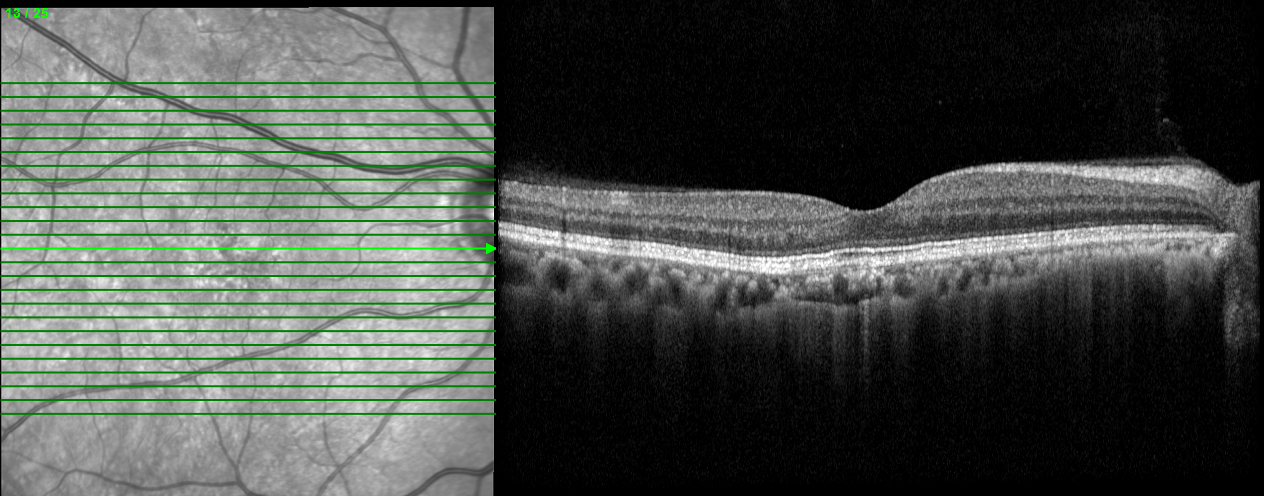


a

b

c

d

e

f

**Supplementary Fig. 3.** Shadow artifact on the en face Haller vessel image resulting from subretinal fluid. (a-c) OCT B-scan images of a patient diagnosed with neovascular age-related macular degeneration. From the left, initial visit, after 3 loading doses of aflibercept, and 1 year after the baseline. (d-f) Structural OCT en face image of a Haller vessel at the same time points as those used for OCT B-scan images. Note that the darker area at the macular area (d, white arrowheads) decreased after 3 loading doses (e) and after 1 year (f). This phenomenon was frequently observed in Haller vessel images at the initial visit, which might represent false vessel parameter values. Therefore, the en face Haller vessel images at the initial visit were not included in this study.


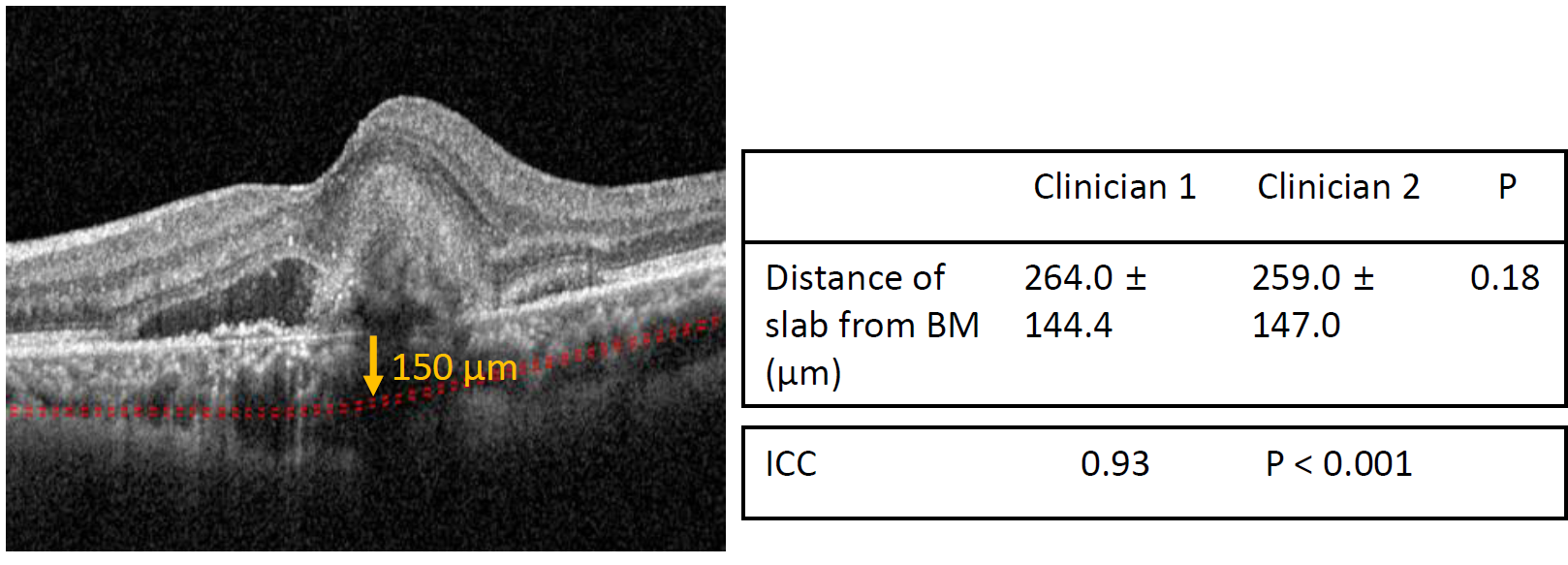
**Supplementary Fig. 4**. Intergrader reliability of the slab location to generate the en face image of the Haller vessel. The en face image of the Haller vessel was generated by locating the slab (10 μm width adjusted from the choriocapillaris slab) passing the middle area of the Haller vessel. To check the intergrader reliability of this method, two retinal specialists (H.C.K. and H.L.) independently determined the location of slab in all included eyes, and the distance from the basement membrane (BM) was recorded (left image). The mean value was compared using the Wilcoxon signed rank test. The intraclass correlation coefficient (ICC) was also calculated to confirm the intergrader reliability. As a result, no difference in slab distance from BM was found (right table), and ICC was sufficiently high to represent the suggested method to generate the consistent en face Haller images among clinicians.

**
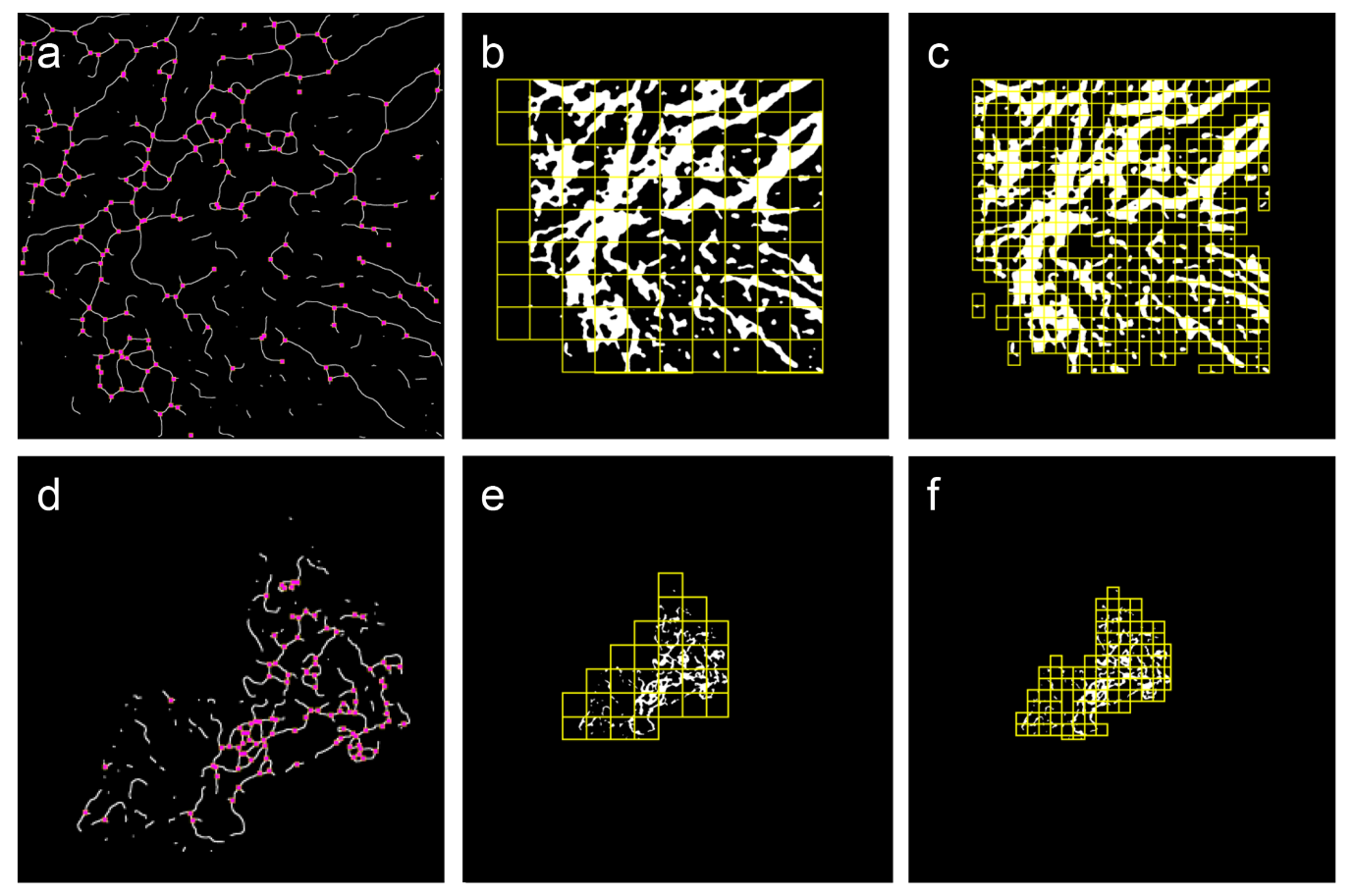
Supplementary Fig. 5** (a) Image after skeletonizing the binarized en face Haller vessel. Using the ‘Analyze skeleton’ function of FIJI, the number of intersections was counted (pink squares). Additionally, the branch vessel lengths were obtained by counting white pixels between different intersections and those between intersections and endpoints when ‘Analyze skeleton’ was applied. Finally, the total vessel length was calculated as the sum of all branch vessel lengths. (b, c) The fractal dimension and lacunarity of each en face Haller vessel image were calculated using the FracLac plugin of FIJI. Based on the information, including the number of boxes (N) covering vessel area according to the diminishing side length of boxes (ε), fractal dimension and lacunarity were calculated. (d, e, f) In MNV, processes to calculate the number of intersections, branch and total vessel lengths, fractal dimension and lacunarity were performed using the same method applied to the en face Haller vessel image.

**Supplementary Table 1.** Baseline characteristics of the included eyes.

| Parameters | Infrequent  (N=20) | Frequent  (N=32) | P value* |  | Visual gain  (N=13) | No visual gain  (N=39) | P value* |
| --- | --- | --- | --- | --- | --- | --- | --- |
| Age | 70.35 ± 9.37 | 70.84 ± 7.85 | 0.93 |  | 70.62 ± 8.06 | 70.67 ± 8.59 | 0.94 |
| Sex (male/female) | 11/9 | 19/13 | 0.04 |  | 9/4 | 21/18 | 0.52 |
| Typical nAMD/PCV | 14/6 | 19/13 | 0.56 |  | 10/3 | 23/16 | 0.33 |
| VA |  |  |  |  |  |  |  |
| Baseline | 0.47 ± 0.61 | 0.61 ± 0.54 | 0.10 |  | 1.20 ± 0.66 | 0.34 ± 0.33 | <0.001 |
| After loading doses | 0.26 ± 0.28 | 0.43 ± 0.40 | 0.10 |  | 0.58 ± 0.42 | 0.29 ± 0.32 | 0.02 |
| At 12 months | 0.23 ± 0.28 | 0.47 ± 0.38 | 0.01 |  | 0.46 ± 0.34 | 0.35 ± 0.37 | 0.20 |
| Number of injections over 12 months | 3.40 ± 0.50 | 6.00 ± 1.05 | <0.001 |  | 4.62 ± 1.26 | 5.13 ± 1.63 | 0.33 |
| SFCT | 266.0 ± 105.8 | 262.5 ± 108.2 | 0.83 |  | 262.2 ± 70.6 | 264.4 ± 70.7 | 0.78 |

Values are expressed as the mean ± standard deviation.

nAMD = neovascular age-related macular degeneration, PCV = polypoidal choroidal vasculopathy, SFCT = subfoveal choroidal thickness

* Mann–Whitney tests were performed for the comparison of the mean values, and the ratio of sex was compared by the chi-square test.

VA, visual acuity.

**Supplementary Table 2.** Quantified morphologic parameters of the Haller vessel and MNV grouped by the number of injections.

|  | Haller vessel | | | |  |  | MNV | | | | | |
| --- | --- | --- | --- | --- | --- | --- | --- | --- | --- | --- | --- | --- |
|  | After loading | | At 12 months | |  |  | Baseline | | After loading | | At 12 months | |
| Parameters | Infrequent  (N=20) | Frequent  (N=32) | Infrequent  (N=12) | Frequent  (N=22) |  | Parameters | Infrequent  (N=20) | Frequent  (N=32) | Infrequent  (N=20) | Frequent  (N=32) | Infrequent  (N=12) | Frequent  (N=22) |
| Diameter |  |  |  |  |  | Diameter |  |  |  |  |  |  |
| Mean | 95.51 ± 13.23 | 100.57 ± 16.73 | 100.52 ± 11.64 | 99.47 ± 16.94 |  | Mean | 20.01 ± 1.51 | 20.57 ± 2.14 | 19.49 ± 1.76 | 20.31 ± 1.99 | 19.96 ± 1.76 | 20.64 ± 1.58 |
| SD | 44.24 ± 11.07 | 53.61 ± 19.40 | 50.77 ± 9.91 | 51.48 ± 16.17 |  | SD | 9.46 ± 1.95 | 9.99 ± 1.71 | 8.70 ± 2.24 | 9.75 ± 2.03 | 9.30 ± 1.91 | 9.95 ± 1.74 |
| Median | 101.53 ± 14.65 | 109.31 ± 18.71 | 105.90 ± 15.26 | 106.59 ± 18.66 |  | Median | 22.93 ± 5.82 | 24.56 ± 4.40 | 20.96 ± 6.58 | 22.93 ± 5.76 | 21.83 ± 6.45 | 24.41 ± 4.60 |
| Maximum | 392.36 ± 83.12 | 413.07 ± 111.86 | 391.92 ± 69.42 | 419.21 ± 87.74 |  | Maximum | 63.54 ± 13.62 | 66.32 ± 12.86 | 59.61 ± 12.37 | 63.86 ± 12.34 | 65.50 ± 18.53 | 67.29 ± 14.74 |
| Total length | 54.05 ± 7.86* | 48.32 ± 7.53* | 52.96 ± 7.52 | 47.86 ± 6.20 |  | Total length | 27.94 ± 37.58 | 30.10 ± 50.88 | 24.20 ± 35.82 | 28.20 ± 42.22 | 39.12 ± 52.41 | 45.07 ± 62.06 |
| No. of intersections | 182.15 ± 42.97 | 161.53 ± 37.96 | 195.75 ± 40.89* | 167.55 ± 36.67* |  | No. of intersections | 340.80 ± 464.60 | 355.34 ± 635.69 | 279.35 ± 464.70 | 328.06 ± 521.98 | 495.25 ± 724.00 | 519.77 ± 756.49 |
| Branch length |  |  |  |  |  | Branch length |  |  |  |  |  |  |
| Mean | 231.33 ± 16.90 | 237.08 ± 27.27 | 227.06 ± 15.89 | 229.71 ± 22.50 |  | Mean | 49.18 ± 4.64* | 51.97 ± 4.85* | 50.30 ± 3.94 | 51.53 ± 5.13 | 52.02 ± 4.99 | 52.40 ± 4.76 |
| SD | 214.39 ± 26.68 | 229.39 ± 44.18 | 208.71 ± 17.70 | 220.95 ± 32.43 |  | SD | 39.34 ± 4.58 | 42.28 ± 5.14 | 42.11 ± 6.77 | 43.19 ± 8.82 | 43.45 ± 7.97 | 44.80 ± 6.87 |
| FD | 1.69 ± 0.04 | 1.68 ± 0.03 | 1.70 ± 0.03* | 1.68 ± 0.03* |  | FD | 1.60 ± 0.12 | 1.60 ± 0.14 | 1.59 ± 0.11 | 1.58 ± 0.16 | 1.61 ± 0.12 | 1.65 ± 0.09 |
| Lacunarity | 0.46 ± 0.09 | 0.48 ± 0.10 | 0.43 ± 0.07 | 0.48 ± 0.09 |  | Lacunarity | 0.56 ± 0.14 | 0.53 ± 0.14 | 0.54 ± 0.09 | 0.53 ± 0.11 | 0.52 ± 0.09 | 0.48 ± 0.07 |
| Vessel area | 11.81 ± 3.00 | 11.67 ± 2.67 | 13.07 ± 2.72 | 11.7 ± 2.40 |  | Vessel area | 0.95 ± 1.32 | 1.05 ± 1.78 | 0.78 ± 1.22 | 0.96 ± 1.52 | 1.42 ± 1.96 | 1.51 ± 2.13 |
| SFCT | 234.15 ± 102.12 | 257.16 ± 116.39 | 247.05 ± 100.51 | 264.88 ± 120.93 |  | MNV area | 3.25 ± 4.27 | 3.62 ± 5.63 | 2.84 ± 4.17 | 3.45 ± 5.60 | 4.41 ± 5.65 | 4.96 ± 6.59 |

Values are expressed as the mean ± SD; unit of vessel area = mm^2^; unit of total vessel length = mm; unit of diameter and branch vessel length = μm. MNV, macular neovascularization; No., number; FD, fractal dimension; SFCT, subfoveal choroidal thickness; SD, standard deviation. * Asterisk was marked when statistical significance (P<.05) was found from the Mann–Whitney test.

**Supplementary Table 3.** Quantified morphologic parameters of MNV and Haller vessel grouped by visual acuity gain.

|  | Haller vessel | | | |  |  | MNV | | | | | |
| --- | --- | --- | --- | --- | --- | --- | --- | --- | --- | --- | --- | --- |
|  | After loading | | At 12 months | |  |  | Baseline | | After loading | | At 12 months | |
| Parameters | Visual gain  (N=13) | No visual gain  (N=39) | Visual gain  (N=8) | No visual gain  (N=26) |  | Parameters | Visual gain  (N=13) | No visual gain  (N=39) | Visual gain  (N=13) | No visual gain  (N=39) | Visual gain  (N=8) | No visual gain  (N=26) |
| Diameter |  |  |  |  |  | Diameter |  |  |  |  |  |  |
| Mean | 98.08 ± 14.00 | 98.80 ± 16.19 | 103.29 ± 14.71 | 98.78 ± 15.34 |  | Mean | 21.05 ± 2.48 | 20.13 ± 1.68 | 20.55 ± 2.26 | 19.81 ± 1.80 | 21.02 ± 1.81 | 20.21 ± 1.59 |
| SD | 49.61 ± 15.96 | 50.14 ± 17.78 | 51.78 ± 12.97 | 51.06 ± 14.69 |  | SD | 10.43 ± 1.54 | 9.57 ± 1.85 | 9.72 ± 2.70 | 9.22 ± 1.97 | 10.25 ± 1.82 | 9.56 ± 1.80 |
| Median | 104.80 ± 16.04 | 106.82 ± 18.17 | 111.35 ± 17.15 | 104.80 ± 17.38 |  | Median | 26.20 ± 0.00 | 23.18 ± 5.59 | 22.17 ± 6.29 | 22.17 ± 6.13 | 24.56 ± 4.63 | 23.18 ± 5.63 |
| Maximum | 396.04 ± 56.39 | 408.13 ± 112.96 | 419.21 ± 42.59 | 406.62 ± 90.95 |  | Maximum | 64.49 ± 8.39 | 65.50 ± 14.41 | 59.46 ± 11.49 | 63.15 ± 12.71 | 65.50 ± 12.13 | 67.01 ± 17.11 |
| Total length | 51.38 ± 7.82 | 50.24 ± 8.25 | 51.24 ± 6.82 | 49.18 ± 7.15 |  | Total length | 20.10 ± 9.60 | 32.32 ± 52.46 | 13.50 ± 8.48 | 31.05 ± 44.71 | 28.43 ± 12.07 | 47.44 ± 65.77 |
| No. of intersections | 169.92 ± 34.06 | 169.31 ± 43.24 | 184.63 ± 43.31 | 175.31 ± 39.57 |  | No. of intersections | 230.69 ± 104.38 | 389.44 ± 654.02 | 141.23 ± 93.84 | 365.36 ± 561.56 | 297.88 ± 119.46 | 576.73 ± 829.77 |
| Branch length |  |  |  |  |  | Branch length |  |  |  |  |  |  |
| Mean | 232.02 ± 19.81 | 235.82 ± 25.15 | 230.03 ± 25.78 | 228.39 ± 18.76 |  | Mean | 53.03 ± 6.23 | 50.18 ± 4.26 | 52.36 ± 7.01 | 50.63 ± 3.66 | 54.84 ± 5.00 | 51.47 ± 4.51 |
| SD | 219.95 ± 30.83 | 224.85 ± 41.41 | 214.44 ± 24.10 | 217.30 ± 30.05 |  | SD | 43.08 ± 6.37 | 40.50 ± 4.51 | 45.75 ± 10.73 | 41.78 ± 6.81 | 48.55 ± 6.93 | 43.02 ± 6.88 |
| FD | 1.68 ± 0.04 | 1.68 ± 0.04 | 1.69 ± 0.03 | 1.69 ± 0.04 |  | FD | 1.61 ± 0.08 | 1.59 ± 0.15 | 1.59 ± 0.08 | 1.58 ± 0.16 | 1.68 ± 0.05 | 1.62 ± 0.11 |
| Lacunarity | 0.47 ± 0.07 | 0.47 ± 0.10 | 0.43 ± 0.06 | 0.47 ± 0.09 |  | Lacunarity | 0.53 ± 0.15 | 0.54 ± 0.14 | 0.51 ± 0.08 | 0.54 ± 0.11 | 0.46 ± 0.07 | 0.51 ± 0.08 |
| Vessel area | 11.69 ± 2.50 | 11.74 ± 2.89 | 12.59 ± 1.82 | 12.06 ± 2.77 |  | Vessel area | 0.70 ± 0.33 | 1.11 ± 1.84 | 0.44 ± 0.30 | 1.04 ± 1.58 | 0.94 ± 0.40 | 1.65 ± 2.31 |
| SFCT | 241.54 ± 120.26 | 250.56 ± 108.85 | 244.46 ± 114.22 | 262.54 ± 113.49 |  | MNV area | 2.47 ± 1.20 | 3.81 ± 5.84 | 1.63 ± 1.04 | 3.74 ± 5.74 | 3.18 ± 1.39 | 5.25 ± 7.00 |

Values are expressed as the mean ± SD. Unit of vessel area = mm^2^; unit of total vessel length = mm; unit of diameter and branch vessel length = μm.

MNV, macular neovascularization; No., number; FD, fractal dimension; SFCT, subfoveal choroidal thickness; VA, visual acuity; SD, standard deviation.

* Statistical significance (P<.05) from Mann–Whitney test.

Due to the limitation of space, exact P values could not be presented, and no statistical significance was found in any comparison between the ‘Visual gain’ and ‘No visual gain’ subgroups.
